# Degeneration in Families: Observations in a Lunatic Asylum

**Published:** 1908-03

**Authors:** 


**Degeneration in Families: Observations in a Lunatic Asylum.** By Fr. Lange, M.D. Authorised Translation from the Danish by C. Chr. Sonne. Pp. 207. London: Henry Kinpton. 1907.

This work contains Dr. Lange's views on the course of degeneration in families, and is based upon a close investigation into the heredity of certain patients received in the Lunatic Asylum at Middelfart, Denmark. His object has been to discover evidence not only of insanity, but of all morbid traits or habits, immoral conduct and defined neuroses, and he has endeavoured to trace the specific results of epileptic, alcoholic and insane heritage in all the members of a family, and not merely in the insane individuals of it. The principal theme is the investigation of the mental phenomena, morbid or otherwise, evidenced in what he calls his great "families," in which, although not clearly stated, he seems to include groups whose history is identifiable on both paternal and maternal sides for four generations. Forty-four of these so-called families have been collected, in which all classes of society, but mostly the educated, have been included. These 44 groups have contributed 70 cases of undoubted insanity to the Middelfart Asylum, whilst there is evidence of 358 other individuals possessing more or less morbid characters; in all, 428 persons showing acknowledged neuropathies. Moreover, in the families contributing the insane patients a considerable number of cases of pronounced insanity has been traced to other asylums. Amongst the members of 23 out of 44 families, actual suicidal attempts were made in 39 cases, sexual perversions found in 8 (probably understatement); alcoholic proclivities were only noted in 18 out of the 44 groups. The author emphasises as a curious fact that only one case of definite criminality was found. His inquiries have taken another turn, that is as to the intellectual value (apart from the pathological relations) of the individuals in the great families. He observed that amongst the 70 persons admitted into the asylum, a comparatively elevated standard of culture and intelligence was notable generally, and dealing with 28 families, whose position in society was such as to allow of intellectual development and its rewards, he found that 72 persons reached high station by reason of their mental ability and culture, and at least an equal number attained more or less widely-recognised success as exponents of art and literature. The general characteristics of the mental disorders amongst the great families seems to be rather that of unstable and oscillating equilibrium, and impulsiveness of the lighter kind than of pronounced or incurable maladies. The author, in search for a common bond or characterising somatic condition, which should unite these families into a group, thinks he has discovered this in a so-called "uratic diathesis or degeneration," either as causal connection, or as a parallel branch from the same trunk; the proof of this diathesis demanded consisting in the unmistakable presence of "gravel" The distinction to be drawn between the mental disorders associated with uratic degeneration and again with those groups of degeneration immediately dependent on alcohol, epilepsy and direct insane inheritance, seems to be that, in these latter, excesses and crime—brutalities, so to speak—alone characterise; in the former or uratic group, good average, even high standard of mental ability had been reached. In the first case it is exemplified in the unripe fruit torn from the tree or untimely rotten; in the second, the final and inevitable decay of a once flourishing tree. The rest of the book consists of general observations on psychology and mental degeneration, which though thoughtful need no special mention here. Notwithstanding that the intimate investigation of long lives of family descent is one which should undoubtedly yield fruitful results, this work lacks any specific scientific value; it is too discursive, too full of general statements which hardly require making, and certainly wait for proof. There is no opportunity for critical comment on the conclusions which the author draws from his observations on his "great families," for it is not obvious of what these consist. In short, much that might be valuable in the author's labour has suffered by the indefinite quasi-colloquial manner of its presentation; interesting indications rather than precise statements result. It must be remembered, however, that Dr. Lange prefers to introduce the work modestly as "observations" rather than as a scientific treatise. We could wish his mode of expression to be less complicated and prolix; the book is often tedious and uneasy reading, and this does not in any way appear to be the fault of the translation, which seems skilfully done.

